# Extended single-dose toxicity study of [^211^At]NaAt in mice for the first-in-human clinical trial of targeted alpha therapy for differentiated thyroid cancer

**DOI:** 10.1007/s12149-021-01612-9

**Published:** 2021-04-19

**Authors:** Tadashi Watabe, Kazuko Kaneda-Nakashima, Kazuhiro Ooe, Yuwei Liu, Kenta Kurimoto, Takashi Murai, Yuka Shidahara, Kenji Okuma, Masanori Takeuchi, Masayuki Nishide, Atsushi Toyoshima, Atsushi Shinohara, Yoshifumi Shirakami

**Affiliations:** 1 Department of Nuclear Medicine and Tracer Kinetics, Graduate School of Medicine, Osaka University, 2-2 Yamadaoka, Suita, Osaka 565-0871 Japan; 2 Institute for Radiation Sciences, Osaka University, Suita, Japan; 3 Core for Medicine and Science Collaborative Research and Education, Project Research Center for Fundamental Sciences, Graduate School of Science, Osaka University, Toyonaka, Japan; 4 Bioscience Business Division, KAC Co., Ltd, Kyoto, Japan; 5 Department of Chemistry, Graduate School of Science, Osaka University, Toyonaka, Japan

**Keywords:** Astatine, Clinical trial, Mouse, Thyroid cancer, Toxicity study

## Abstract

**Objective:**

Astatine (^211^At) is a promising alpha emitter as an alternative to iodine (^131^I). We are preparing the first-in-human (FIH) clinical trial of targeted alpha therapy for differentiated thyroid cancer in consultation with Pharmaceuticals and Medical Devices Agency. Here, we performed an extended single-dose toxicity examination under a reliability standard, as a preclinical safety assessment of [^211^At]NaAt to determine the FIH dose.

**Methods:**

[^211^At]NaAt solution was injected into normal 6-week-old mice (male (*n* = 50) and female (*n* = 50), body weight: male 33.2 ± 1.7 g, female 27.3 ± 1.5 g), which were then divided into four groups: 5 MBq/kg (*n* = 20), 20 MBq/kg (*n* = 20), 50 MBq/kg (*n* = 30), saline control (*n* = 30). The mice were followed up for 5 days (primary evaluation point for acute toxicity: *n* = 80) or 14 days (*n* = 20: evaluation point for recovery) to monitor general condition and body weight change. At the end of the observation period, necropsy, blood test, organ weight measurement, and histopathological examination were performed. For body weight, blood test, and organ weight, statistical analyses were performed to compare data between the control and injected groups.

**Results:**

No abnormal findings were observed in the general condition of mice. In the 50 MBq/kg group, males (days 3 and 5) showed a significant decrease in body weight compared with the control. However, necropsy did not differ significantly beyond the range of spontaneous lesions. In the blood test, males (50 MBq/kg) and females (50 MBq/kg) showed a decrease in white blood cell and platelet counts on day 5, and recovery on day 14. In the testis, a considerable weight decrease was observed on day 14 (50 MBq/kg), and multinucleated giant cells were observed in all mice, indicating a significant change related to the administration of [^211^At]NaAt.

**Conclusions:**

In the extended single-dose toxicity study of [^211^At]NaAt, administration of high doses resulted in weight loss, transient bone marrow suppression, and pathological changes in the testis, which require consideration in the FIH clinical trial.

## Introduction

In recent years, radionuclide therapy is attracting attention for the treatment of various types of cancers. Among them, targeted alpha therapy with an alpha emitter is promising, with higher therapeutic effect than conventional beta radionuclide therapy [1]. Astatine (^211^At) is an alpha emitter that can be produced by the ^209^Bi(α, 2n)^211^At reaction using cyclotron [2]. Recently, we reported the excellent anti-tumor effect of [^211^At]sodium astatide ([^211^At]NaAt) in a preclinical study using a xenograft model of differentiated thyroid cancer [2]. We are now preparing for an investigator-initiated clinical trial of targeted alpha therapy for differentiated thyroid cancer refractory to standard ^131^I treatment or tyrosine kinase inhibitor, in consultation with the Pharmaceuticals and Medical Devices Agency (PMDA), the Japanese authority for drug approval. As there is no report on the intravenous administration of [^211^At]NaAt in humans, it is necessary to carefully perform a preclinical safety assessment for the first-in-human (FIH) clinical trial. Fortunately, alpha therapy using ^223^Ra (Xofigo) has already been approved in Japan, and its safety information can be used as a reference, to some extent [3]. However, as there is no description of radiopharmaceuticals in the official guidelines (ICH M3), which can be used as an official reference for PMDA, it was necessary to determine the optimum evaluation time point with reference to published reports with adequate evidence [4–6].

In addition, there is no preclinical safety testing in association with GLP facilities capable of handling alpha-emitting nuclides in Japan. Thus, a toxicity study must be performed in our institution under the reliability standard based on Article 43 of the Japanese Pharmaceutical Law Enforcement Regulations with the support of contractors with adequate GLP compliance experience. As the permitted amount of ^211^At in preclinical radioactive controlled areas is limited by the Radiation Hazard Prevention Act, it is difficult to conduct toxicity studies in rats, dogs, and monkeys. In consultation with the PMDA, it was determined that the [^211^At]NaAt clinical trial can begin on the premise that it should be carried out with extreme caution so long as no obvious safety issues arise during the extended single-dose toxicity study in mice. Hence, the current study is an extended single-dose toxicity examination using mice with reference to the ICH guidelines and previous reports, which was performed in accordance with the reliability standard as a preclinical safety assessment of [^211^At]NaAt to determine the FIH dose [4–7].

## Materials and methods

### Test substance

Sodium astatide ([^211^At]NaAt), which contains ^211^At^−^ (astatide ion) at a concentration of 1.5–15.0 MBq/mL at the time of preparation (radionuclide purity > 99.99%, radiochemical purity: 93.4 ± 1.1%), was used. The stability of [^211^At]NaAt was confirmed for at least 7 h. The storage temperature was 23–27 °C. Saline (Japanese Pharmacopeia Saline, Otsuka, Japan) was used as the injection medium. The stock solution of sodium astatide ([^211^At]NaAt) was diluted with saline to a volume of approximately 100 µL.

### Animal preparation and administration

This study was conducted in compliance with the Animal Experiment Regulations and the Act on Welfare and Management of Animals of Osaka University Graduate School of Medicine (approval number: No. 30-103-004). Normal specific-pathogen-free ICR mice (5–6 weeks old) were purchased from Japan SLC Inc. (Hamamatsu, Japan). A total of 122 mice (61 males and 61 females) were obtained, 100 of which (50 males and 50 females) were used in the experiment. The general condition of the mice was visually confirmed at the time of administration, and animals without any abnormalities were housed in the breeding chamber. The mice were housed under a 12 h light/12 h dark cycle [chamber temperature: 23 °C (range: 20.9–25.6 °C)], with ad libitum access to food and water. The acclimatization period was approximately 1 week before administration of the test substance. Daily observation of general condition and measurement of body weight were performed, which were used as indices for selecting test animals on the day of test substance administration. The body weight ranges in male and female mice at the time of administration were 28.9–35.2 and 23.0–30.5 g, respectively.

The ICH M3 (R2) guideline was used as a reference to determine the required number of mice for each group: 10 mice/sex/group for the main evaluation point (all groups) and 5 mice/sex for recovery evaluation (selected group) [4]. With respect to the evaluation time point, the next day after treatment is the main evaluation time point in the ICH M3 (R2) guideline. However, as the effects of radiation appear slightly delayed, 5 days after the administration was selected as the primary evaluation point, after referencing a previous report [6]. The dose was decided with reference to the effective dose (5 MBq/kg) in a previous study [2].

Three doses of the test substance (5, 20, and 50 MBq/kg, single-dose) or saline were intravenously administered to 20 mice (10 males and 10 females) per dose group for 5 days. In addition, 10 mice (5 males and 5 females) were administered the test substance (50 MBq/kg) or saline for 14 days.

### Animal observation

General conditions (behavior, appearance, and function) were observed for 5 or 14 days after treatment. The observations were made immediately after administration of the test substance, 1, 2, and 3 h after administration, and every morning, afternoon, and evening in the later days until day 5 or day 14. Body weight was monitored at the time of administration (day 0) and 3, 5, 10, and 14 days after administration (days 3, 5, 10, and 14).

### Blood examination

The mice were fasted overnight (approximately 16 h or more) from the evening of the day before blood collection. Under isoflurane inhalation anesthesia, blood was collected from the abdominal aorta using a needle-syringe with sodium heparin as the anticoagulant on days 5 and 14. A blood cell counter was used for measurements (CB-1010; ARKRAY, Inc. Kyoto, Japan). Red blood cell count (RBC), hemoglobin concentration (Hb), hematocrit (Ht), mean corpuscular volume (MCV), mean corpuscular hemoglobin (MCH), mean corpuscular hemoglobin concentration (MCHC), white blood cell count (WBC), platelet count (platelet), lymphocytes (%Lymph), monocytes (%Mon), and granulocytes (%Gra) were calculated. As the WBC was very low (0.5 × 10^3^ cells/μL or less) in one male in the 20 MBq/kg group and three males in the 50 MBq/kg group, their lymphocyte, monocyte, and granulocyte levels were not calculated.

Plasma was obtained by centrifuging a portion of the blood sample collected at the time of hematological testing (4 °C, 230 G, 10 min) and measured using a dry clinical chemistry analyzer (Spotchem D-00 QR D-02; ARKRAY, Inc. Kyoto, Japan). The levels of aspartate aminotransferase (AST), alanine aminotransferase (ALT), gamma-glutamyl transpeptidase (γ-GTP), lactate dehydrogenase (LDH), alkaline phosphatase (ALP), creatine kinase (CK), amylase (AMY), total bilirubin (TBIL), creatinine (CRE), urea nitrogen (BUN), glucose (GLU), total protein (TP), albumin (ALB), total cholesterol (TCHO), triglyceride (TG), and electrolytes (Na, K, and Cl) were measured. For one male mouse in the 5 MBq/kg administration group, the amount of blood collected was too low, and plasma could not be obtained. In addition, γ-GTP, LDH, CK, and AMY values below or above the measurement range were treated as reference values and excluded from the calculation of mean and standard deviation.

### Necropsy and organ weight measurement

On day 5 or 14, the mice under isoflurane inhalation anesthesia were euthanized by cutting the abdominal aorta and posterior vena cava after collecting blood. The mice were dissected according to a pathological procedure, and a macroscopic examination of the external surface, skull, thoracic cavity, abdominal cavity, and their contents, was performed. The excised organs and tissues included the brain, pituitary gland, tongue, trachea, thyroid gland (including epithelial body), esophagus, salivary gland (submandibular gland/submandibular gland), thymus, heart, lung (including bronchus), liver and gallbladder, pancreas, stomach, small intestine, large intestine, mesenteric lymph node, spleen, kidney, adrenal gland, bladder, testis, upper body of testis, seminal vesicle, prostate, ovary, uterus, vagina, skin, mammary gland, spinal cord, bone and bone marrow (femoral bone), eyeballs and adrenal glands, carcasses, and other organs and tissues with macroscopic changes. The brain, salivary glands (bilateral sublingual gland/submandibular gland), heart, lung (bilateral), liver and gallbladder, spleen, kidney (bilateral), testis (bilateral), and ovary (bilateral) were weighed according to the ICH S4 guidelines [8].

### Histological evaluation

The excised organs and tissues were fixed in a 10% neutral buffered formalin solution, the testes were fixed in a mixed solution of formalin/sucrose/acetic acid, and the eyeballs were fixed in an eye fixative (Superfix, Kurabo Industries Ltd. Osaka, Japan). After fixation, paraffin sections were prepared and stained with hematoxylin and eosin (HE). For staining, an automatic dyeing device (Tissue Tech DRS2000-B; Sakura Fine Tech Japan Co., Ltd. Tokyo, Japan) was used. For the control and 50 MBq/kg group, the brain (cerebrum), thyroid gland (including epithelial body), trachea, esophagus, salivary gland (sublingual gland/submandibular gland), thymus, heart, lung (including bronchi), liver and gallbladder, pancreas, stomach, jejunum, duodenum, colon, mesenteric lymph node, spleen, kidney, adrenal gland, bladder, testis, ovary, uterus, bone and bone marrow (femoral bone), eyeballs, as well as other organs and tissues with macroscopic changes, were examined. For the 5 and 20 MBq/kg groups, the thyroid glands, salivary glands, stomach, duodenum, spleen, kidneys, testes, ovaries, and other organs with macroscopic changes were examined. In addition, other organs/tissues with changes considered to be related to test substance administration in the 50 MBq/kg group were examined. Specimens were evaluated using an integrated fluorescence microscope (BZ-X810; Keyence Corporation. Osaka, Japan).

### Statistical analysis

For each group, the mean and standard deviation (mean ± SD) of weight, blood test results, and organ weights (absolute and relative weights) were calculated. The mean values of the control group and each administration group were compared using Dunnett’s test. Statistical analyses were carried out using SPSS (version 19.0), and statistical significance was set at *p* < 0.05.

## Results

### General conditions

**Fig. 1:**
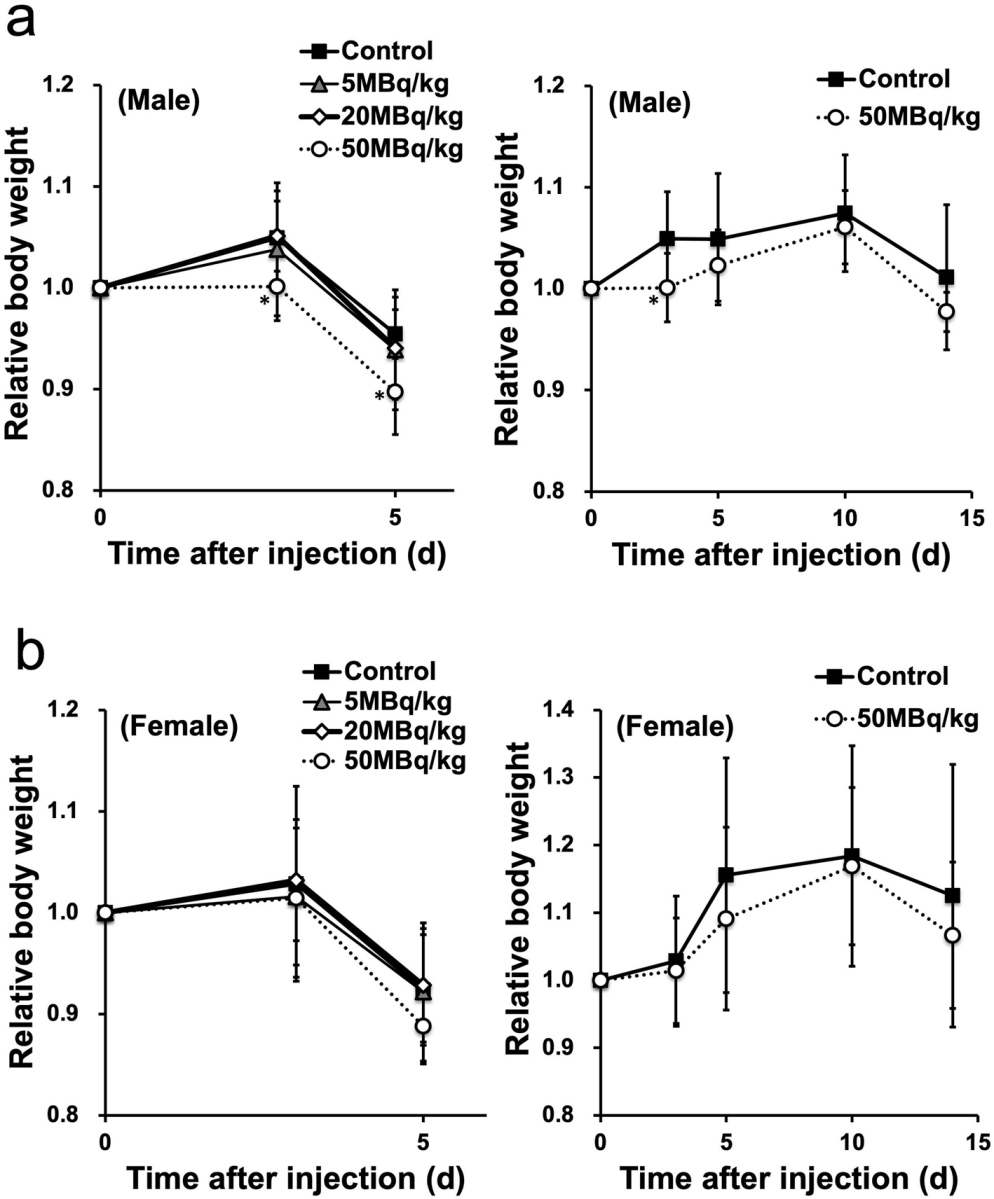
Relative changes in body weight after a single intravenous administration of [^211^At]NaAt in **a** males and **b** females (*p < 0.05 compared with the control group using Dunnett’s test). Note that the last measurement on days 5 and 14 was affected by fasting overnight from the evening of day 4 or day 13 for the blood examination, respectively

In the evaluation of general condition, no deaths or abnormalities were observed in any mice during the observation period (5 or 14 days after administration). A significant decrease in body weight was observed on days 3 and 5 in the male 50 MBq/kg group (5 days monitoring; *p* = 0.016 and *p* = 0.021, respectively) and on day 3 in the male 50 MBq/kg group (14 days monitoring; *p* = 0.003) compared to that of the control group (Fig. 1).

### Blood examination

The hematological test results showed a significant decrease in WBC count in the male 20 MBq/kg group (*p* = 0.033), and in WBC and platelet counts in the 50 MBq/kg group (both male and female) on day 5 compared to the corresponding values in the control group (*p* = 0.012 and *p* < 0.001 for male, and *p* = 0.007 and *p* < 0.001 for female, respectively; Table 1). These significant decreases in the WBC and platelet counts were recovered on day 14. On day 5, TG in the male 5 MBq/kg and 20 MBq/kg groups, LDH in the 50 MBq/kg group, and ALP and TG in the female 50 MBq/kg group showed a significant decrease (*p* = 0.036, *p* = 0.010, *p* = 0.008, *p* = 0.024, and *p* = 0.034, respectively), while Na in the male 50 MBq/kg group and TCHO in the female 20 MBq/kg group showed a significant increase (*p* = 0.038 and *p* = 0.049, respectively) compared to the corresponding values in the control group. On day 14, AST and TBIL in the male 50 MBq/kg group, and AST and AMY in the female 50 MBq/kg group showed a significant decrease (*p* = 0.001, *p* = 0.030, *p* = 0.011, and *p* = 0.009, respectively), while TP, ALB, and TCHO in males and females in the 50 MBq/kg group showed a significant increase (*p* = 0.028, *p* = 0.023, and *p* = 0.002 for male; *p* = 0.001, *p* = 0.028, and *p* < 0.001 for female, respectively) compared to the corresponding values in the control group (Table 2). However, no abnormal findings were observed in the related organs or tissues (liver and kidney). Thus, we could not determine whether the change was caused by the administration of the test substance.

**Table 1:** Hematology—expanded single dose intravenous toxicity study of ^211^At-NaAt in mice –

Inspection time	Sex	Group		RBC	Hb	Ht	MCV	MCH	MCHC	WBC	Platelet	%Lymph	%Mon	%Gra
				(× 10^6^cell/μL)	(g/dL)	(%)	(fL)	(pg)	(g/dL)	(× 10^3^cell/μL)	(× 10^3^cell/μL)	(%)	(%)	(%)
5 days after administration	Male	Control	N	10	10	10	10	10	10	10	10	10	10	10
			Mean	8.48	14.5	44.1	52.1	17.1	32.9	6.9	1300.2	64.8	4.3	31.0
			± S.D	± 0.54	± 1.0	± 3.1	± 1.4	± 0.5	± 0.6	± 5.4	± 480.7	± 17.9	± 4.2	± 14.9
		5 MBq/kg	N	10	10	10	10	10	10	10	10	10	10	10
			Mean	8.13	14.1	42.4	52.3	17.4	33.3	4.6	1091.5	66.3	4.2	29.6
			± S.D	± 1.20	± 1.8	± 6.1	± 0.8	± 0.6	± 1.0	± 3.6	± 315.5	± 15.2	± 3.6	± 13.0
		20 MBq/kg	N	10	10	10	10	10	10	10	10	9	9	9
			Mean	8.30	14.4	43.3	52.2	17.3	33.3	2.4*	1036.8	73.3	2.5	24.2
			± S.D	± 0.35	± 0.7	± 2.4	± 1.1	± 0.5	± 0.6	± 3.4	± 301.8	± 9.1	± 1.1	± 8.8
		50 MBq/kg	N	10	10	10	10	10	10	10	10	7	7	7
			Mean	8.11	13.8	42.1	51.9	17.0	32.8	1.6*	623.7*	70.1	3.6	26.3
			± S.D	± 1.00	± 1.8	± 5.3	± 1.0	± 0.4	± 0.3	± 2.3	± 314.0	± 12.8	± 1.6	± 12.0
5 days after administration	Female	Control	N	10	10	10	10	10	10	10	10	10	10	10
			Mean	8.60	15.1	45.1	52.5	17.5	33.3	5.3	1003.5	62.8	3.8	33.4
			± S.D	± 0.42	± 0.8	± 2.3	± 1.2	± 0.3	± 0.4	± 3.1	± 198.4	± 13.6	± 3.5	± 10.8
		5 MBq/kg	N	10	10	10	10	10	10	10	10	10	10	10
			Mean	8.78	15.2	45.9	52.4	17.3	33.1	4.7	1105.3	69.1	2.5	28.4
			± S.D	± 0.58	± 1.0	± 2.8	± 1.8	± 0.8	± 0.5	± 2.5	± 143.0	± 4.7	± 0.7	± 4.4
		20 MBq/kg	N	10	10	10	10	10	10	10	10	10	10	10
			Mean	8.90	15.4	46.4	52.2	17.3	33.2	3.2	913.3	68.4	2.9	28.7
			± S.D	± 0.31	± 0.4	± 1.7	± 2.0	± 0.6	± 0.4	± 3.4	± 236.8	± 9.5	± 1.9	± 9.0
		50 MBq/kg	N	10	10	10	10	10	10	10	10	7	7	7
			Mean	8.53	15.1	44.8	52.5	17.6	33.6	1.3*	613.5*	64.6	3.7	31.6
			± S.D	± 0.31	± 0.8	± 2.3	± 1.5	± 0.5	± 0.4	± 1.5	± 176.1	± 19.8	± 2.1	± 18.2
14 days after administration	Male	Control	N	5	5	5	5	5	5	5	5	5	5	5
			Mean	8.41	14.5	42.9	51.0	17.2	33.8	4.8	1139.2	69.5	2.4	28.1
			± S.D	± 0.73	± 1.7	± 4.0	± 0.6	± 0.5	± 0.8	± 3.7	± 187.4	± 11.4	± 2.0	± 9.4
		50 MBq/kg	N	5	5	5	5	5	5	5	5	5	5	5
			Mean	7.76	13.7	40.7	52.5	17.7	33.7	4.3	1028.6	44.6*	4.7	50.8*
			± S.D	± 0.32	± 0.7	± 2.4	± 1.4	± 0.4	± 0.6	± 3.1	± 75.7	± 11.1	± 2.6	± 9.6
	Female	Control	N	5	5	5	5	5	5	5	5	5	5	5
			Mean	8.90	15.1	45.5	51.3	17.0	33.2	6.3	1396.3	72.3	3.2	24.6
			± S.D	± 0.50	± 0.5	± 1.0	± 2.6	± 0.5	± 0.8	± 3.9	± 346.0	± 14.6	± 3.2	± 11.4
		50 MBq/kg	N	5	5	5	5	5	5	5	5	5	5	5
			Mean	8.73	15.1	44.8	51.4	17.2	33.6	5.0	1085.0	61.0	3.4	35.6
			± S.D	± 0.23	± 0.4	± 1.7	± 1.2	± 0.4	± 0.5	± 2.8	± 73.2	± 9.7	± 1.3	± 9.0

*RBC* Red blood cell, *Hb* Hemoglobin, *Ht* Hematocrit, *MCV* Mean corpuscular volume, *Gra* Granulocyte, *WBC* White blood cell, *MCH* Mean corpuscular hemoglobin level, *Lym* Lymphocyte, *MCHC* Mean corpuscular hemoglobin concentration, *Mon* Monocyte

Significantly different from the control, *p<0.05. (Dunnett-test)

**Table 2:** Blood chemistry—expanded single dose intravenous toxicity study of ^211^At-NaAt in mice -

Inspection time	Sex	Group		AST	ALT	γ-GTP	LDH	ALP	CK	AMY	TBIL	CRE
				(IU/L)	(IU/L)	(IU/L)	(IU/L)	(IU/L)	(IU/L)	(IU/L)	(mg/dL)	(mg/dL)
5 days after administration	Male	Control	N	10	8	0	8	10	8	10	10	3
			Mean	55	18	–	1047	368	353	2908	0.59	0.27
			± S.D	± 22	± 7	–	± 459	± 79	± 266	± 435	± 0.25	± 0.06
		5 MBq/kg	N	8	7	0	8	9	9	9	8	0
			Mean	47	24	–	1170	356	394	2578	0.58	–
			± S.D	± 20	± 15	–	± 271	± 129	± 327	± 547	± 0.25	–
		20 MBq/kg	N	9	8	0	6	10	9	10	9	1
			Mean	56	32	–	880	335	407	2853	0.59	0.20
			± S.D	± 31	± 41	–	± 329	± 85	± 176	± 417	± 0.24	–
		50 MBq/kg	N	10	9	0	9	10	10	8	10	0
			Mean	43	20	–	546**	309	344	3019	0.60	–
			± S.D	± 23	± 10	–	± 123	± 59	± 194	± 361	± 0.26	–

Inspection time	Sex	Group		BUN	GLU	TP	ALB	TCHO	TG	Na	K	Cl
				(mg/dL)	(mg/dL)	(mg/dL)	(mg/dL)	(mg/dL)	(mg/dL)	(mg/dL)	(mg/dL)	(mg/dL)
5 days after administration	Male	Control	N	10	10	10	10	10	10	10	10	10
			Mean	39.8	180	4.1	2.1	141	123	142	5.0	120
			± S.D	± 8.6	± 43	± 0.3	± 0.2	± 18	± 22	± 2	± 0.7	± 3
		5 MBq/kg	N	9	9	9	9	9	9	9	8	9
			Mean	39.0	150	4.3	2.2	123	90*	144	4.8	122
			± S.D	± 7.7	± 44	± 0.2	± 0.1	± 14	± 23	± 3	± 0.6	± 4
		20 MBq/kg	N	10	10	10	10	10	10	10	9	10
			Mean	40.2	156	4.4	2.3	139	85**	144	5.0	122
			± S.D	± 10.6	± 31	± 0.3	± 0.2	± 22	± 19	± 2	± 0.7	± 4
		50 MBq/kg	N	10	10	10	10	10	10	10	10	10
			Mean	36.5	162	4.4	2.2	132	96	145*	4.9	121
			± S.D	± 7.8	± 44	± 0.5	± 0.1	± 21	± 40	± 4	± 1.0	± 4

Inspection time	Sex	Group		AST	ALT	γ-GTP	LDH	ALP	CK	AMY	TBIL	CRE
				(IU/L)	(IU/L)	(IU/L)	(IU/L)	(IU/L)	(IU/L)	(IU/L)	(mg/dL)	(mg/dL)
5 days after administration	Female	Control	N	10	4	0	10	10	10	10	10	0
			Mean	41	16	–	795	377	315	2757	0.66	-
			± S.D	± 16	± 4	–	± 354	± 83	± 283	± 431	± 0.32	-
		5 MBq/kg	N	10	2	0	10	10	10	9	10	0
			Mean	37	15	–	754	376	265	2770	0.66	-
			± S.D	± 11	± 3	–	± 391	± 75	± 220	± 165	± 0.23	-
		20 MBq/kg	N	9	4	0	9	10	10	10	9	1
			Mean	35	14	–	704	374	594	2792	0.64	0.80
			± S.D	± 7	± 6	–	± 262	± 70	± 542	± 400	± 0.28	-
		50 MBq/kg	N	9	4	0	9	10	10	9	9	0
			Mean	28	18	–	508	287*	450	2474	0.57	-
			± S.D	± 10	± 8	–	± 169	± 61	± 447	± 356	± 0.26	-

Inspection time	Sex	Group		BUN	GLU	TP	ALB	TCHO	TG	Na	K	Cl
				(mg/dL)	(mg/dL)	(mg/dL)	(mg/dL)	(mg/dL)	(mg/dL)	(mg/dL)	(mg/dL)	(mg/dL)
5 days after administration	Female	Control	N	10	10	10	10	10	10	10	10	10
			Mean	27.6	159	4.1	2.2	95	84	145	5.1	116
			±S.D.	±5.7	±38	±0.3	±0.1	±15	±29	±4	±0.7	±4
		5 MBq/kg	N	10	10	10	10	10	10	10	10	10
			Mean	32.2	156	4.0	2.2	107	77	146	5.0	116
			±S.D.	±5.9	±41	±0.4	±0.2	±18	±18	±6	±0.7	±3
		20 MBq/kg	N	10	10	10	10	10	10	10	9	10
			Mean	31.9	161	4.2	2.3	117*	78	144	5.1	117
			±S.D.	±7.3	±25	±0.3	±0.2	±27	±23	±3	±0.8	±3
		50 MBq/kg	N	10	10	10	10	10	10	10	9	10
			Mean	34.7	165	4.2	2.2	114	56*	145	4.5	119
			±S.D.	±9.7	±37	±0.4	±0.2	±15	±24	±3	±0.5	±3

Inspection time	Sex	Group		AST	ALT	γ-GTP	LDH	ALP	CK	AMY	TBIL	CRE
				(IU/L)	(IU/L)	(IU/L)	(IU/L)	(IU/L)	(IU/L)	(IU/L)	(mg/dL)	(mg/dL)
14 days after administration	Male	Control	N	5	5	0	4	5	3	5	5	4
			Mean	56	20	–	998	244	617	2655	0.80	0.30
			±S.D.	±8	±6	–	±262	±43	±436	±397	±0.41	±0.00
		50 MBq/kg	N	5	3	0	5	1	5	5	5	0
			Mean	31**	14	–	694	370	365	2612	0.32*	–
			±S.D.	±8	±2	–	±363	–	±188	±334	±0.04	–
	Female	Control	N	5	3	0	5	5	5	5	5	1
			Mean	46	14	–	976	281	544	2341	0.52	0.20
			±S.D.	±7	±2	–	±439	±76	±420	±73	±0.18	–
		50 MBq/kg	N	5	2	0	5	2	5	5	5	0
			Mean	33*	13	–	711	166	214	2071**	0.48	–
			±S.D.	±4	±1	–	±273	±17	±141	±163	±0.11	–

Inspection time	Sex	Group		BUN	GLU	TP	ALB	TCHO	TG	Na	K	Cl
				(mg/dL)	(mg/dL)	(g/dL)	(g/dL)	(mg/dL)	(mg/dL)	(mEq/L)	(mEq/L)	(mEq/L)
14 days after administration	Male	Control	N	5	5	5	5	5	5	5	5	5
			Mean	52.0	177	4.1	1.9	130	74	140	4.6	123
			±S.D.	±20.0	±38	±0.3	±0.1	±28	±13	±3	±0.7	±1
		50 MBq/kg	N	5	5	5	5	5	5	5	5	5
			Mean	33.8	198	4.6*	2.2*	196**	75	140	4.2	123
			±S.D.	±6.5	±17	±0.3	±0.2	±14	±17	±3	±0.5	±6
	Female	Control	N	5	5	5	5	5	5	5	5	5
			Mean	29.0	164	4.1	2.1	111	73	144	4.6	120
			±S.D.	±4.4	±61	±0.1	±0.1	±14	±13	±2	±1.3	±1
		50 MBq/kg	N	5	5	5	5	5	5	5	5	5
			Mean	22.6	204	4.8**	2.4*	171**	64	145	3.9	119
			±S.D.	±5.4	±27	±0.3	±0.2	±17	±17	±2	±1.1	±2

*AST* Aspartate aminotransferase, *ALT* Alanine aminotransferase, *γ-GTP* γ-glutamyl transpeptidase, *LDH* Lactate dehydrogenase, *ALP* Alkaline phosphatase, *CK* Creatine kinase, *AMY* Amylase, *TBIL* Total bilirubin, *CRE* Creatinine, *BUN* Blood urea nitrogen, *ALB* Albumin, *GLU* Glucose, *TP *Total protein, *TG* Triglyceride, *TCHO* Total cholesterol, *Na* Sodium, *K* Potassium, *Cl* Chlorine

Significantly different from the control, **p < 0.01 (Dunnett-test)

Significantly different from the control, *p < 0.05, **p < 0.01 (Dunnett-test)

### Necropsy and organ weight measurement

Necropsy revealed no abnormal findings that could be attributed to the administration of the test substance (Table 3). There were some cases of abdominal black-red mass, small spleen, and small testis, which were considered spontaneous lesions.

**Table 3:** Necropsy findings—expanded single dose intravenous toxicity study of ^211^At-NaAt in mice -

Inspection time											5 days after administration								
Sex											Male					Female			
Group											Control	5 MBq/kg	20 MBq/kg	50 MBq/kg		Control	5 MBq/kg	20 MBq/kg	50 MBq/kg
No. of animals											10	10	10	10		10	10	10	10
No. of surviving animals											10	10	10	10		10	10	10	10
No. of dead animals											0	0	0	0		0	0	0	0
No. of no abnormality											10	8	10	8		10	9	10	10
Organ				Necropsy findings															
Spleen				: Small in size							0	0	0	1		0	0	0	0
Testis				: Small in size (Left)							0	0	0	1		–	–	–	–
Epididymis				: Reddish color of the cauda epididymis (Right)							0	1	0	0		–	–	–	–
Abdominal cavity				: Black-red mass 3 mm in diameter							0	1	0	0		0	1	0	0
				in the mesentery of the liver															

Inspection time	14 days after administration			
Sex	Male		Female	
Group	Control	50 MBq/kg	Control	50 MBq/kg
No. of animals	5	5	5	5
No. of surviving animals	5	5	5	5
No. of dead animals	0	0	0	0
No. of no abnormality	5	5	5	5

**Fig. 2:**
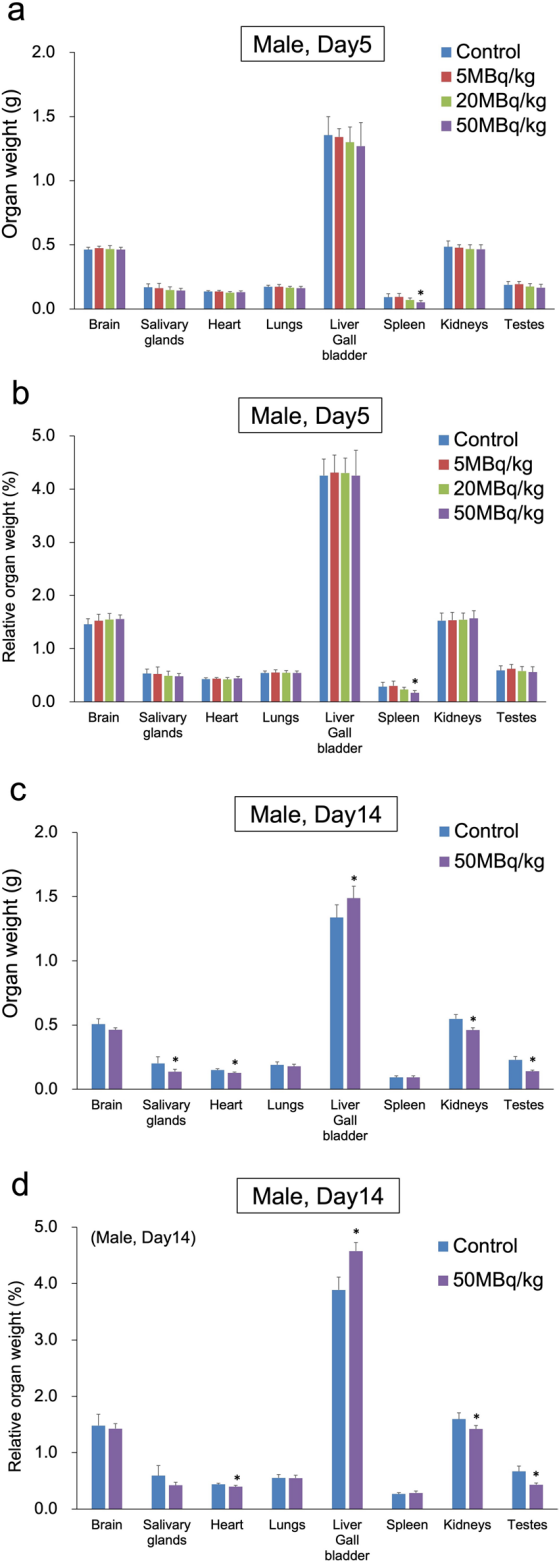
Comparison of organ weight after a single intravenous administration of [^211^At]NaAt in males: **a** absolute organ weight on day 5, **b** relative organ weight on day 5, **c** absolute organ weight on day 14, **d** relative organ weight on day 14 (*p < 0.05 compared with the control group using Dunnett’s test)

**Fig. 3:**
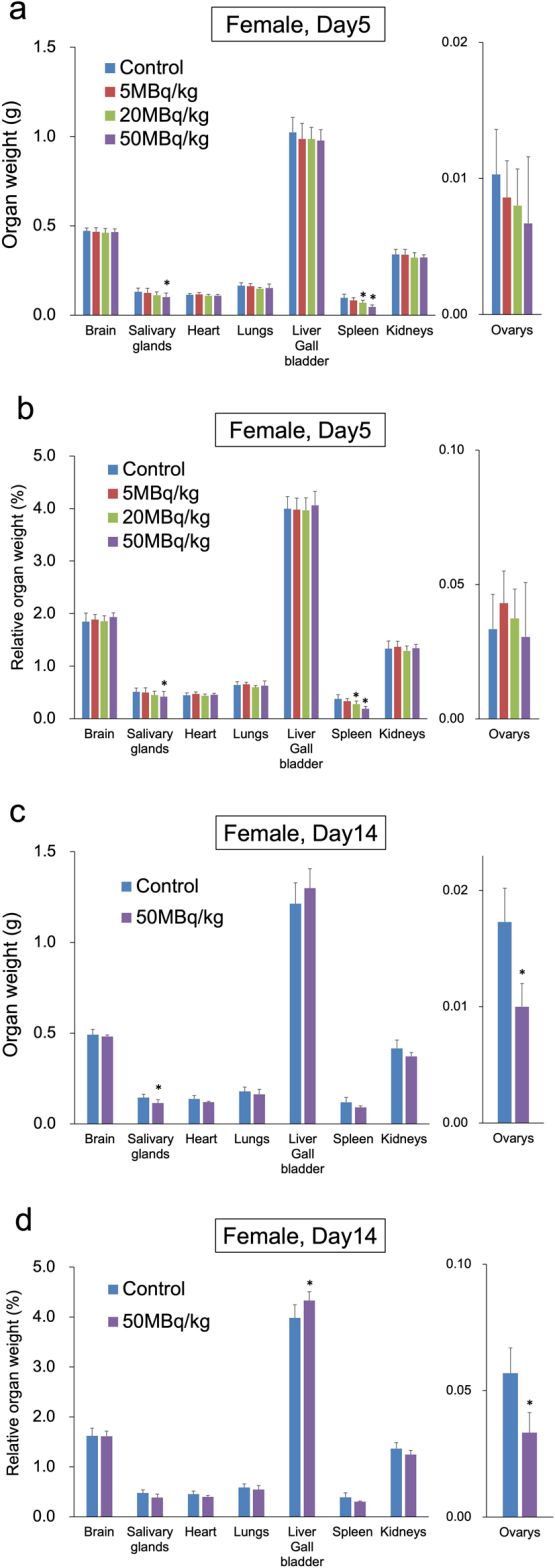
Comparison of organ weight after a single intravenous administration of [^211^At]NaAt in females: **a** absolute organ weight on day 5, **b** relative organ weight on day 5, **c** absolute organ weight on day 14, **d** relative organ weight on day 14 (*p < 0.05 compared with the control group using Dunnett’s test)

The organ weight results are shown in Figs. 2 and 3. On day 14, the testes showed a significant weight decrease in the 50 MBq/kg group compared to that in the control group (*p* < 0.001), with the histopathological examination revealing abnormal findings, indicating that the change was due to the administration of the test substance. On day 5, a dose-dependent, significant decrease, or decreasing trend, was observed in the spleen weight of the 20 and 50 MBq/kg groups (male and female) compared to that in the control group. The salivary gland also showed a significant weight decrease in the female 50 MBq/kg group compared to that in the control group (*p* = 0.011), and a non-significant, however, dose-dependent decrease in the female 5 and 20 MBq/kg groups. Similar findings were observed in the salivary glands of males. On day 14, a significant weight decrease in the salivary gland, heart, kidney, adrenal gland, and ovary, as well as a significant weight increase in the liver was observed in the 50 MBq/kg group compared to the organ weights in the control group (*p* = 0.031, *p* = 0.002, *p* = 0.001, *p* = 0.018, *p* = 0.002, and *p* = 0.037, respectively). However, there were no related abnormalities in the histopathological examination of these organs, and the effect of the test substance was unclear.

### Histological evaluation

**Fig. 4:**
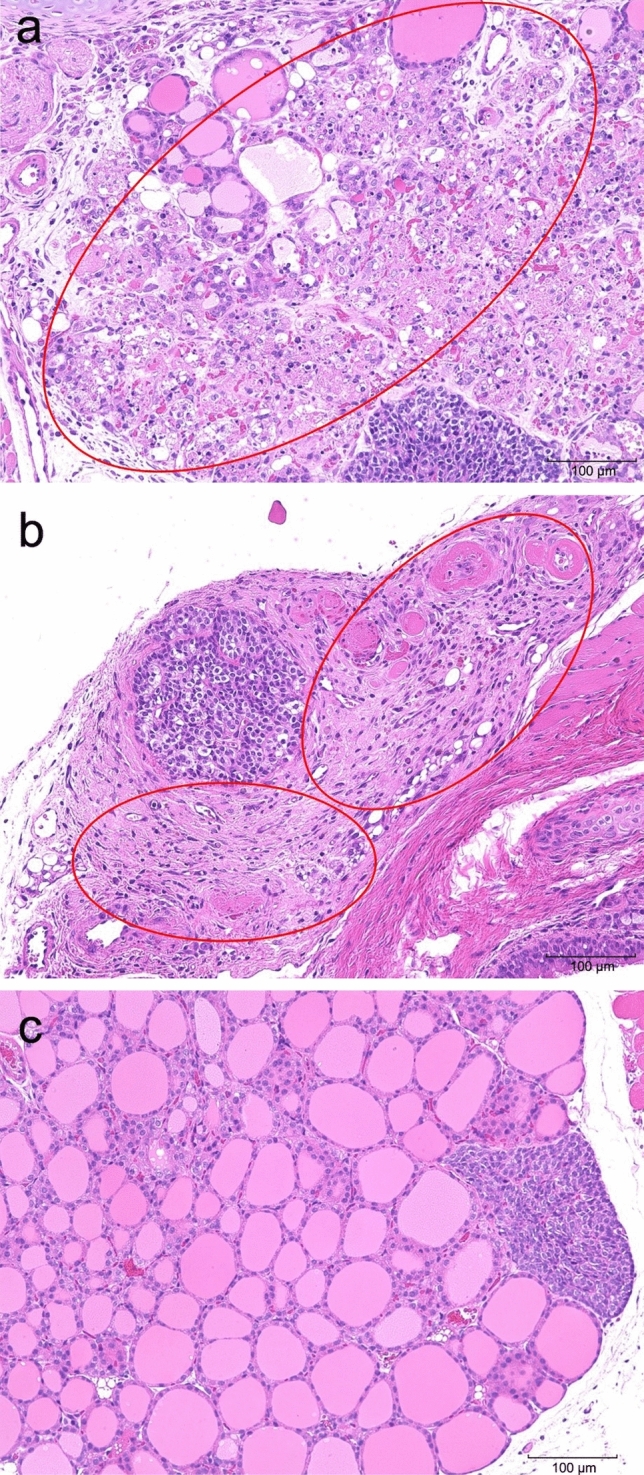
HE-stained section of the thyroid after a single intravenous administration of [^211^At]NaAt (magnification × 200). **a** Thyroid and parathyroid on day 5 (male, 50 MBq/kg). Colloidal decrease in the thyroid gland, degeneration/necrosis of follicular epithelium, and infiltration of inflammatory cells were observed (red circled area). **b** Thyroid and parathyroid on day 14 (male, 50 MBq/kg). Colloidal decrease in the thyroid gland, fibrosis, and inflammatory cell infiltration were observed (red circled areas). **c** Normal thyroid and parathyroid in the control group (male, day 5)

**Fig. 5:**
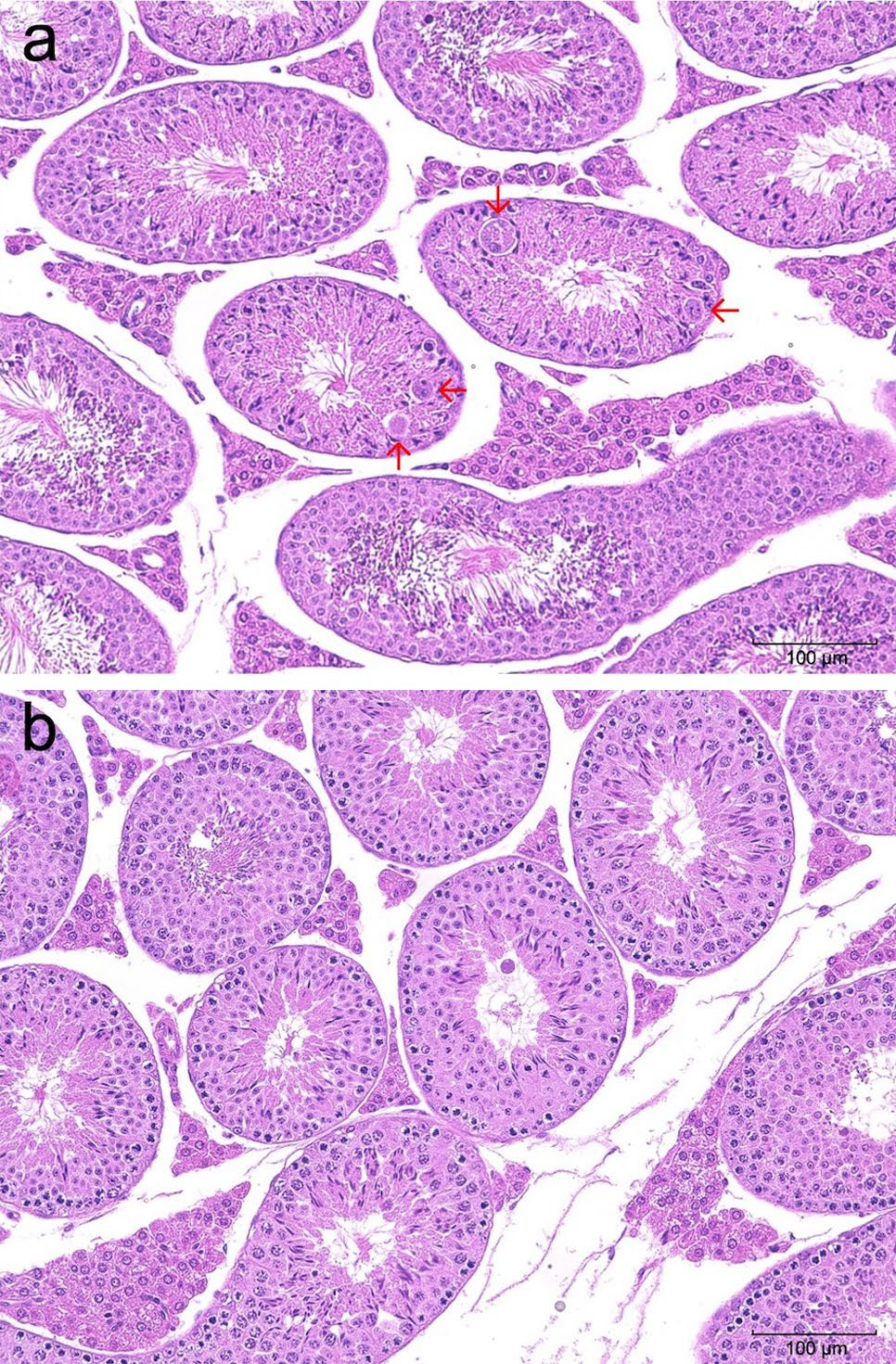
HE-stained section of the testis after a single intravenous administration of [^211^At]NaAt (magnification × 200). **a** Testis from the 50 MBq/kg group mouse on day 14. The appearance of multinucleated giant cells (red arrows) was observed in the fine tubes. **b** Normal testis in the control group (day 14)

**Fig. 6:**
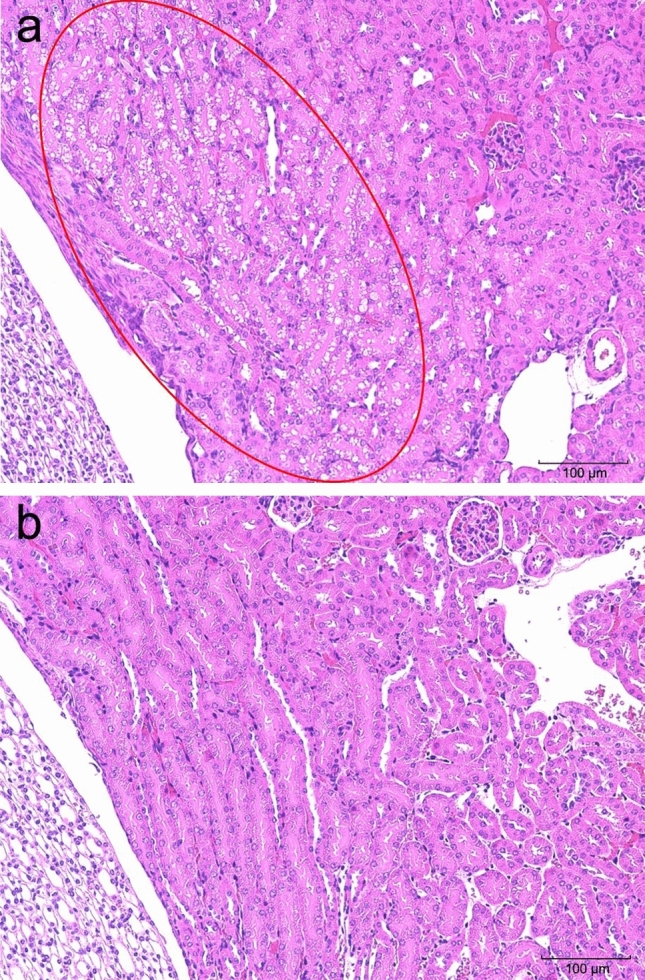
HE-stained section of the kidney after a single intravenous administration of [^211^At]NaAt (magnification × 200). **a** Kidney from the 50 MBq/kg group mouse on day 5 (female). Tubular degeneration was observed in the tubular epithelium of the outer medulla (red circle area). **b** Normal kidney in the control group (female, day 5)

Table 4 shows the results of histopathological examination. Colloidal decrease, degeneration/necrosis of follicular epithelial cells, and inflammatory cell infiltration were observed as dose-dependent changes in the thyroid gland on days 5 and 14, indicating the well-known ablation effect (Fig. 4). On day 14, multinucleated giant cells were observed in the testes of the 50 MBq/kg group, not in the control group, indicating that the change was due to the administration of the test substance (Fig. 5). In addition, vacuolization in the renal tubules of the outer medulla was observed in the kidneys of the female 50 MBq/kg group on days 5 and 14 (Fig. 6). Squamous metaplasia, glandular dilatation, and inflammatory cell infiltration was observed in the glandular stomach on days 5 and 14. Moreover, degeneration/necrosis, mineral deposition, and yellowish-brown pigmentation and fibrosis in the cortex and medulla of the adrenal gland in the male 50 MBq/kg group; sperm stasis and hypoplasia in the testes of the 5 and 50 MBq/kg groups; as well as degeneration/necrosis of the epididymal epithelium, fibrosis, and interstitial mononuclear cell infiltration in the epididymis of the 5 MBq/kg group, were observed on day 14. However, these findings were considered spontaneous lesions as they were not dose-dependent and some were also observed in the control group, however, in only a small number of cases. No abnormalities were found in the histological analyses of the other organs.

**Table 4:** Histopathological findings—expanded single dose intravenous toxicity study of ^211^At-NaAt in mice –

Inspection time		5 days after administration																															
Sex		Male																															
Group		Control									5 MBq/kg					20 MBq/kg									50 MBq/kg								
No. of animals		10									10					10									10								
Grade of lesion		*N*		±		+		++		+ + +	*N*	±	+	+ +	+ + +	*N*		±		+		+ +		+ + +	*N*		±		+		+ +		+ + +
Organ	Histopathological findings																																
Thyroid	: Decreased colloid	9		0		1		0		0	6	0	3	0	1	0		0		5		4		1	1		0		1		1		7
	: Degenration/necrosis, follicular cell	9		0		1		0		0	7	0	2	0	1	0		0		9		0		1	1		0		1		3		5
	: Infiltrate, inflammatory cell, diffuse	10		0		0		0		0	8	1	0	1	0	0		6		3		1		0	1		1		2		6		0
Stomach	: Cyst, glandular, squamous metaplasia	10		0		0		0		0	9	0	1	0	0	9		0		1		0		0	10		0		0		0		0
	: Dilatation, gland	9		0		1		0		0	8	0	2	0	0	7		0		3		0		0	8		0		2		0		0
	: Infiltrate, inflammatory cell, focal	10		0		0		0		0	9	0	1	0	0	10		0		0		0		0	10		0		0		0		0
Kidney	: Cast, hyaline	9		0		1		0		0	10	0	0	0	0	10		0		0		0		0	10		0		0		0		0
	: Regenaration, tubule	8		0		1		1		0	10	0	0	0	0	10		0		0		0		0	10		0		0		0		0
Testis	: Hypoplasia	10		0		0		0		0	10	0	0	0	0	10		0		0		0		0	9		0		0		1		0
	: Sperm stasis, focal	10		0		0		0		0	10	0	0	0	0	9		0		1		0		0	10		0		0		0		0
Epididymis^*1^	: Degeneration/necrosis, epithelial, focal	Not examined									0	0	1	0	0	Not examined									Not examined								
	: Fibrosis, focal										0	0	1	0	0																		
	: Infiltrate, inflammatory cell, focal										0	1	0	0	0																		
Intraperitoneal nodule^*1^	: Congestion	Not examined									0	0	1	0	0	Not examined									Not examined								
	: Fibrous capsule										0	0	1	0	0																		
	: Necrotic liver mass										0	0	1	0	0																		

Inspection time		5 days after administration																																			
Sex		Female																																			
Group		Control									5 MBq/kg									20 MBq/kg									50 MBq/kg								
No. of animals		10									10									10									10								
Grade of lesion		*N*		±		+		++		+++	*N*		±		+		++		+++	*N*		±		+		++		+++	*N*		±		+		++		+++
Organ	Histopathological findings																																				
Thyroid	: Decreased colloid	10		0		0		0		0	10		0		0		0		0	0		0		8		2		0	0		0		0		6		4
	: Degenration/necrosis, follicular cell	10		0		0		0		0	10		0		0		0		0	0		0		9		1		0	0		0		1		5		4
	: Infiltrate, inflammatory cell, diffuse	10		0		0		0		0	10		0		0		0		0	0		5		5		0		0	0		0		6		4		0
Stomach	: Cyst, glandular, squamous metaplasia	10		0		0		0		0	9		0		1		0		0	9		0		1		0		0	10		0		0		0		0
	: Dilatation, gland	9		0		1		0		0	6		0		4		0		0	9		0		1		0		0	6		0		4		0		0
	: Infiltrate, inflammatory cell, focal	10		0		0		0		0	10		0		0		0		0	10		0		0		0		0	9		0		1		0		0
Kidney	: Vacuolation, tubule, outer medulla	10		0		0		0		0	10		0		0		0		0	10		0		0		0		0	7		0		3		0		0
Adrenal	: Accessory adrenocortical tissue	10		0		0		0		0	Not examined									Not examined									9		0		1		0		0
Intraperitoneal nodule*^1^	: Congestion	Not examined									0		0		1		0		0	Not examined									Not examined								
	: Fibrous capsule										0		0		1		0		0																		
	: Necrotic liver mass										0		0		1		0		0																		

Inspection time		14 days after administration																			
Sex		Male										Female									
Group		Control					50 MBq/kg					Control					50 MBq/kg				
No. of animals		5					5					5					5				
Grade of lesion		*N*	±	+	++	+++	*N*	±	+	++	+++	*N*	±	+	++	+++	*N*	±	+	++	+++
Organ	Histopathological findings																				
Thyroid	: Decreased colloid	5	0	0	0	0	0	0	0	0	5	5	0	0	0	0	0	0	0	0	5
	: Fibrosis	5	0	0	0	0	0	0	0	0	5	5	0	0	0	0	0	0	0	0	5
	: Infiltrate, inflammatory cell, diffuse	5	0	0	0	0	0	0	5	0	0	5	0	0	0	0	0	0	4	1	0
Stomach	: Cyst, glandular, squamous metaplasia	5	0	0	0	0	5	0	0	0	0	3	0	2	0	0	4	0	1	0	0
	: Dilatation, gland	2	0	3	0	0	1	0	4	0	0	5	0	0	0	0	3	0	2	0	0
Kidney	: Vacuolation, tubule, outer medulla	5	0	0	0	0	5	0	0	0	0	5	0	0	0	0	4	0	1	0	0
Adrenal	: Degenration/necrosis, focal, cortex and medulla	5	0	0	0	0	4	0	0	1	0	5	0	0	0	0	5	0	0	0	0
	: Fibrosis, cortex	5	0	0	0	0	4	0	1	0	0	5	0	0	0	0	5	0	0	0	0
	: Mineralization, focal, cortex and medulla	5	0	0	0	0	4	0	1	0	0	5	0	0	0	0	5	0	0	0	0
	: Pigmentation, yellowish brown, cortex	5	0	0	0	0	4	0	1	0	0	5	0	0	0	0	5	0	0	0	0
Testis	: Multinucleated giant cell	5	0	0	0	0	0	0	5	0	0										

No remarkable changes were seen in the brain, parathyroid, trachea, esophagus, salivary gland (submandibular gland), salivary gland (sublingual gland), sublingual gland, thymus, heart, lung, bronchia, liver, gall bladder, pancreas, duodenum, jejunum, colon, mesenteric lymph node, spleen, adrenal, urinary bladder, femur, bone marrow and eye

Grade of lesion : *N* Normal,* ±* Minimal or abnormality, + Slight, *++ *Moderate, *+++* Severe

*1 *N*=1 (5 MBq/kg)

## Discussion

In the extended single-dose toxicity study of [^211^At]NaAt using mice conducted under a reliability standard, administration of [^211^At]NaAt at high doses (50 MBq/kg) resulted in weight loss, transient bone marrow suppression, and pathological changes in the testis. In the evaluation of body weight change, the 50 MBq/kg group showed a decreasing tendency from days 3 to 5 after administration, as confirmed in a previous study [5]. Blood examination revealed a transient decrease in WBC and platelet counts was observed in the 50 MBq/kg group (males and females) on day 5 compared to those of the control group, however, they recovered on day 14. This result was also consistent with that of a previous study [5]. In addition, TP, ALB, and TCHO were significantly higher in the male and female 50 MBq/kg groups on day 14 than in the control group, which was confirmed in previous studies and considered to reflect hypothyroidism via thyroid ablation [5]. Meanwhile, the decrease in TG (day 5 in 5 and 20 MBq/kg male, and 50 MBq/kg female), which was transiently observed followed by subsequent recovery on day 14, may have been related to the acute lipid metabolism disorder caused by hypothyroidism. In addition, the pathological significance of decreased LDH (day 5, 50 MBq/kg male), AST and TBIL (day 14, 50 MBq/kg male), AST and AMY (day 14, 50 MBq/kg female) and ALP (day 5, 50 MBq/kg female) is unknown as an abnormality or dysfunction is generally considered when these values are significantly elevated beyond the normal range.

In the organ weight measurement, the decrease in spleen weight on day 5, which was primarily observed in the 50 MBq/kg group, may be due to the effect of [^211^At] NaAt, as it is an organ with high physiological accumulation [5]. The weight of the salivary glands also tended to decrease in a dose-dependent manner, mainly at the concentration of 50 MBq/kg, however, no related abnormalities were detected in the histopathological examination. Moreover, considering that the physiological accumulation of [^211^At]NaAt and [^131^I]NaI treatment reportedly exerts adverse effects on the salivary gland, such as swelling, dysgeusia, and decreased salivary secretion, caution must be exercised when conducting clinical trials for adverse effects on the salivary glands. Meanwhile, the testes showed a significant weight decrease in the 50 MBq/kg group on day 14, and histopathological changes (appearance of multinucleated giant cells) were considered to be the effect of [^211^At]NaAt. However, no clear histological finding was observed in spermatogenesis compared to the control group. Since the formation of multinucleated giant cells was relatively mild, it is considered that these changes were not significant enough to induce histological changes in spermatogenesis. Although no significant abnormality was observed in the ovary, there was a significant weight decrease in the 50 MBq/kg group on day 14. As the reproductive organs are highly radiosensitive among the major organs, it is necessary to pay particular attention to fertility, and patients who desire to preserve fertility should be excluded from the clinical trial of [^211^At]NaAt. Furthermore, the decrease in heart weight on day 14 may represent a secondary effect of the hypometabolism or lipid metabolism disorder which caused the change in TCHO by thyroid ablation, as radiation-induced toxicity is uncommon in the heart. Alternatively, the observed decrease in kidney weight on day 14 must be monitored for potential radiation-induced toxicity; however, this may have also been a secondary effect as no significant corresponding histopathological changes were observed. In fact, the reference standard for evaluation of toxicity is histopathological examination. Thus, considering that these changes in weight were minimal, they might reflect other conditions or metabolic changes induced by thyroid ablation.

Histopathological examination showed colloidal depletion, degeneration/necrosis of follicular epithelial cells, and inflammatory cell infiltration in the thyroid gland on days 5 and 14, and there are well-known ablation effects. In addition, squamous epithelial cysts, gastric gland dilatation, and inflammatory cell infiltration were observed in the stomach on days 5 and 14, however, these changes were considered spontaneous lesions as they were not dose-dependent and were also observed in the control group. On days 5 and 14, vacuolation in the renal tubules of the outer renal medullary layer was observed in the kidneys of the female 50 MBq/kg group. Although vacuolation was observed only in the high-dose group, it has been reported in the renal tubules of the outer medullary layer in ICR mice [9]. In addition, as the findings on day 14 were similar to those on day 5 and no necrotic changes were observed on day 5, it was considered that the change was spontaneous. On day 5, the accessory adrenocortical tissue was observed under the adrenal capsule in the adrenal glands of the female 5 MBq/kg group, which was considered to be a spontaneous lesion as it is known as a tissue dysplasia during the embryonic period in mice [10]. In addition, on day 14, degeneration/necrosis, mineral deposits, multinucleated giant cells, yellowish-brown pigmentation, and fibrosis were observed in the adrenal cortex of one mouse in the male 50 MBq/kg group. As it was accompanied by chronic changes such as mineral deposition and fibrosis, and only one male case presented these symptoms on day 14, it was considered to be a spontaneous lesion. On day 5, sperm stasis and hypoplasia were observed in one mouse of the 5 and 50 MBq/kg groups, however, sperm stasis was observed only in the 20 MBq/kg group. Testicular hypoplasia was considered a spontaneous lesion as it was likely to have occurred before the administration of the test substance considering the degree of testicular hypoplasia was strong despite no necrotic changes. In addition, the testicular weight of the 50 MBq/kg group on day 5, which showed testicular hypoplasia, did not differ significantly from that of the control group, and is, therefore, considered to be spontaneous in combination with histological findings. On day 5, the epididymal tail was red in one mouse in the 5 MBq/kg group at necropsy, and degeneration/necrosis of the epididymal epithelium, fibrosis, and interstitial mononuclear cell infiltration were observed. This change was observed in only one mouse in the low-dose group and did not occur in the higher dose groups, suggesting that it was a spontaneous lesion unrelated to test substance administration. An intra-abdominal mass was found in one male and one female in the 5 MBq/kg group by necropsy on day 5. In mice, it is known that the entire lobe can be infarcted due to torsion of the lobe base of the caudate lobe [11], and the intra-abdominal mass observed in this study may have been formed in association with the infarction. As it occurred in only one case in each group and did not occur in the higher dose groups, it was considered a spontaneous lesion unrelated to administration.

Regarding the comparison of absorbed doses in each organ, which was calculated in our previous study, the highest absorbed dose was observed in the thyroid gland (5691 mGy/MBq), followed by the stomach (3941 mGy/MBq) and bladder (1955 mGy/MBq) [5]. Those pertaining to the thyroid agree with the current study results, as the ablation effect was observed in all groups (> 5 MBq/kg). In the stomach, accumulation of ^211^At is responsible for the parietal cells to secrete gastric acid into the gastric juice [2, 5]. Considering that this secretion also includes ^211^At, and is cleared from the stomach wall into the stomach contents, the direct radiation effect in the stomach wall is not considered to be significant, considering the short range of alpha ray in the stomach contents. Alternatively, the testes were considered to be radiosensitive based on the observed histological abnormalities of the high-dose group (50 MBq/kg), as the absorbed dose was reported to be relatively low (83.3 mGy/MBq) in our previous study [5].

As described above, the approximate lethal dose of [^211^At]NaAt exceeds 50 MBq/kg under the conditions of this study. No observable adverse effect level (NOAEL) was considered to be less than 5 MBq/kg as the histopathological examination showed the ablation effect of [^211^At]NaAt in the thyroid gland of the 5 MBq/kg group. However, no abnormal findings related to the administration of [^211^At]NaAt, with the exception of the thyroid, was observed in the 5 MBq/kg group. As inclusion criteria for the FIH clinical trial will include patients with differentiated thyroid cancer who have undergone total thyroidectomy, these individuals will not be affected by thyroid toxicity. In selecting the FIH dose, we will use a common approach to set a start dose at 1/10 the severely toxic dose in 10% of the animals (STD 10) as described in the ICH S9 guidelines [12]. Since the STD 10 of [^211^At]NaAt is > 50 MBq/kg from the results of this study, it is reasonable to use 5 MBq/kg in mice as a reference to set the FIH dose.

## Conclusions

In the extended single-dose toxicity study of [^211^At]NaAt conducted under the reliability standard, no abnormal findings were obtained compared with those of previous studies. Treatment with [^211^At]NaAt (50 MBq/kg) at high doses resulted in weight loss, transient bone marrow suppression, and pathological changes in the testis, which require consideration in the FIH clinical trial.
